# Hydrate of Chloral

**Published:** 1872-05

**Authors:** C. P. Whitehead

**Affiliations:** Lake Providence, La.


### HYDRATE OF CHLORAL.

BY C. P. WHITEHEAD, M.D., LAKE PROVIDENCE, LA.

In the Atlanta Medical and Surgical Journal (number for March, 1872), under the heading, "Professional Olla-Podrida," is an article on hydrate of chloral. As my views in regard to the use of this drug are somewhat different from those of the author of said article, I will, with your permission, state briefly a few points of difference.

I will state, as a premise, that the drug I have prescribed was dispensed by a druggist who has been doing business at his present location for thirty years—is a graduate of one of the best pharmaceutical colleges in America, and enjoys an enviable reputation for probity.

Instead of prescribing it in simple water, I have generally ordered it in syrup of tolu. C. Lewis Diehl, of Louisville, Kentucky, proposes the following, which I deem an admirable vehicle for the conveyance of hydrate of chloral, as well as many other medicines:

℞.—Oil of orange fʒj; oil of cinnamon M. x; oil of anise M. iv; oil of bitter almond M. ij; tincture of cardamom fʒx; stronger alcohol O. ij; cocoa ℥j; carb. magnesiæ ʒij; water O. ivss; sugar lb. iij. M. S. A. utfit elixir. To this add chloral ʒj to elixir ℥j. The *modus operandi* for manufacturing simple elixir will be found in the *Richmond and Louisville Medical Journal*—number for April, 1872.

I have given much more heroic doses than Dr. Battey; never gave so small a dose as seven and one-half grains to an adult; have given four grains to a child seven years old.

I believe, with the Doctor, that the day for table-spoonful doses of calomel has passed, and that we should administer only so much of a medicine as we deem necessary to produce promptly the desired effect.

Probably cases may be met with where seven and a half grain doses are indicated, but I have not met such a case as yet. The *Philadelphia Medical and Surgical Reporter* (Vol. xxv., pp. 501–5) publishes an article upon the drug in question, in which the author states that he gave twenty grain doses for two hundred and fifty-seven nights, with the happiest effect; and in another, sixty grains for one hundred and ninety-five nights. Same volume, page 572, sixty grains are given two or three times per hour.

Judging from the best authorities that have come to my notice, it seems to me, that if a good article of chloral is obtained, and care taken to observe that grave lung or other disease does not contraindicate its use, we do the drug an injustice to class it among the list of dangerous remedies, or to give it in too small doses to secure its full and happy effect.

I frequently prescribe it for the violent headaches accompanying disordered menses. In such cases, I give it at night, generally, in doses of thirty grains, and always procure for my patient eight or ten hours good sleep, followed by no disagreeable symptoms. I am afraid if I were to give fractional doses, sound sleep would not be produced, and consequently nature would not have that opportunity for recuperation as when given in large doses?

I have given one hundred and twenty grains a day in *mania a potu.*

In a case of threatened premature labor—where the patient was suffering terribly from bearing-down pains—thinking it impossible to avoid the catastrophe, and circumstances preventing my staying with the patient, I ordered thirty grains of hydrate of chloral, to be repeated in thirty minutes, if pain continued unrelieved. I afterward learned that the second dose caused all pains to cease, and three weeks subsequently, she was delivered of a healthy child.

---

Analysis of Melolontha Vulgaris.—The cockchafer or May-bug contains, according to an analysis by Dr. Schreiner, considerable quantities of oxalate of lime, uric acid and urates, some leucin, sarkin, and indistinct traces of xanthin, and melolonthin, a crystalizable body of the composition $C_5H_{12}N_2SO_3$.—*Annal. d. Chem. u. Ph., March,* 1872, 252–262.

